# Efficacy of acupuncture for persistent and intractable hiccups: A protocol for systematic review and meta-analysis of randomized controlled trials

**DOI:** 10.1097/MD.0000000000024879

**Published:** 2021-02-26

**Authors:** Yu Zhang, Xudong Jiang, Zhijie Wang, Mingming He, Zimeng Lv, Qing Yuan, Weixun Qin

**Affiliations:** a The First Affiliated Hospital of Anhui University of Chinese Medicine, Hefei; b Shanxi Province Hospital of Traditional Chinese Medical, Taiyuan; c The Fourth Department of Acupuncture and Moxibustion, Shaanxi Traditional Chinese Medicine Hospital, Xi’an; d Guangzhou University of Chinese Medicine, Guangzhou, China.

**Keywords:** acupuncture, meta-analysis, persistent and intractable hiccups, protocol, systematic review

## Abstract

**Background:**

Persistent and intractable hiccups are a common clinical symptom that cause considerable physical pain to patients and severely damage their quality of lives. An increasing number of studies have demonstrated that acupuncture applied at acupoints dominated by Cuanzhu (BL2) can be used as one of the nonpharmacological therapies for controlling intractable hiccups. However, there is insufficient evidence evaluating the safety and effectiveness of those interventions. Therefore, this study is intended to conduct a systematic review and meta-analysis to provide evidence for a further study investigating alternative treatment options for persistent and intractable hiccups.

**Methods and Analysis:**

Randomized controlled trials (RCTs) of adult patients aged >18 years who meet the criteria for intractable hiccup diagnosis will be included, regardless of gender, nationality, and education level. Eight electronic databases will be searched, including 4 Chinese databases (CNKI, SinoMed, Wanfang Database, and Chinese Scientific Journal Database), 4 English databases (Web of Science, Medline, Embase, and Cochrane Library), from their date of establishment to September 2020. Two independent reviewers will evaluate the title summary for each RCT. Disagreements will be discussed with a third commentator. Data integration, heterogeneity analysis, subgroup analysis, and sensitivity analysis, will be performed using R-3.3.2 software. The RevMan 5.3 software will be used for the meta-analysis, and the “risk of bias” assessment will be conducted based on the methodological quality of the included trials recommended by the Cochrane Handbook 5.1. The quality evaluation of this study will be completed by the Grading of Recommendations, Assessment, Development, and Evaluation (GRADE).

**Results:**

This study will summarize all the selected trials aimed at estimating the effectiveness, as well as safety, of applying acupuncture at acupoints dominated by Cuanzhu (BL2) to persistent and intractable hiccups.

**Conclusions:**

This systematic review will provide evidence to assess the validity and safety of applying acupuncture at acupoints dominated by Cuanzhu (BL2) for persistent and intractable hiccups, which may provide clinicians with more choices in the treatment of this disease.

**PROSPERO registration number:**

CRD42020114900.

## 1 Introduction

### 1.1 Description of the condition

Hiccups are caused by the stimulation of the phrenic nerve and the vagus nerve, and the diaphragm and intercostal muscles are involuntarily synchronized with strong rhythmic contraction, causing the throat to produce a short, loud sound. A retrospective study reported that 84 patients were diagnosed with hiccups in Kirikkale University Medical Faculty Emergency Department from 2013 to 2018. Duration of hiccups attack was <48 hours in 44.1% of patients, between 48 hours and 1 month (persistent) in 36.9%, and longer than 1 month (intractable) in 19%.^[1]^ Based on duration, hiccups are classified as intractable that last for more than 30 days.^[2]^ The incidence of intractable hiccups is relatively high when combined with certain underlying disorders such as Parkinson's disease, stroke, advanced tumors, and gastroesophageal reflux disease.^[3–5]^ Studies have reported that approximately 10% of patients with gastroesophageal reflux disease and 20% of patients with Parkinson's disease may develop a hiccup at the time of consultation, whereas only 3% of individuals without these conditions could develop a hiccup.^[6,7]^ In addition to causing severe discomfort and pain, intractable hiccups can cause attention deficit, malnutrition, and weight loss, leading to fatigue, insomnia, anxiety, depression, and reduced quality of life of the patient.^[8,9]^

Clinically, a variety of different medical drugs are used to treat intractable hiccups, such as diazepam, quinine, chlorpromazine, metoclopramide, and baclofen;^[10]^ however, the evidence related to the use of these drugs has been primarily derived from case reports, which necessitates further studies to confirm their effectiveness. In cases where drug treatments are ineffective, it has been reported that surgical methods can be used to relieve the compression.^[11]^ Moreover, alternative treatment options such as hypnosis, breath holding, and swallowing dry granulated sugar have also been proposed to control intractable hiccups.^[12]^ However, drugs and surgical treatments may result in varying degrees of adverse reactions, and alternative therapies lack high-quality evidence to support their efficacy.

Acupoints dominated by Cuanzhu (BL2) for controlling persistent and intractable hiccups has been widely used in alternative treatment methods and confirmed to be more effective than other therapies.^[13–19]^ However, there is no systematic review and meta-analysis to evaluate the potential benefits and harms of applying acupuncture at acupoints dominated by Cuanzhu (BL2) in the treatment of intractable hiccups. Therefore, in case this treatment proves to be effective, we could obtain a rapid, safe, and effective method to help patients relieve their conditions.

### 1.2 Description of the intervention

Numerous clinical trials have demonstrated that acupuncture can treat intractable hiccups, including acupressure, acupoint injection, electroacupuncture, and acupoint embedding.^[13–19]^ Previous studies have also suggested that acupuncture is effective as an adjunct replacement therapy for intractable hiccups after stroke.^[20]^ A systematic review of Cochrane included 4 RCTs evaluating the effectiveness of different acupuncture treatments for intractable hiccups.^[21]^ Although all 4 studies suggested that acupuncture is effective in the treatment of intractable hiccups, it is unfortunate that none of the 4 studies provided a placebo control group, nor did they explain the side effects and complications.

**Figure 1:**
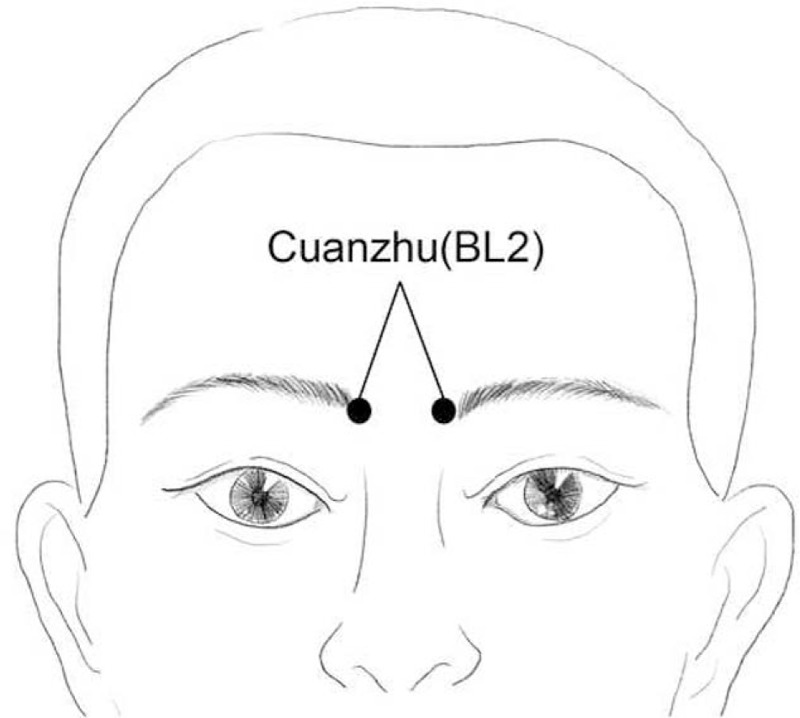
Location of Cuanzhu (BL2).

Cuanzhu (BL2) is an acupoint located on the medial end of the eyebrow (Fig. 1). We selected Cuanzhu as it belongs to the Bladder Meridian of Foot-Taiyang and has the effect of lowering the adverse qi (a disorder of qi leads to hiccups according to the traditional Chinese medicine theory). It is the empirical point in Chinese medicine acupuncture treatment for hiccups. Studies have reported that the total effective rates were 96.4% in the treatment group (using both thumbs to press the 2 Cuanzhu to treat the hiccups, with the power ranging from light to heavy for 2–3 minutes and the patient taking a deep breath and holding their breath for >30 second) and 81.5% in the control group (using baclofen).^[22]^ Another study reported that based on conventional treatment, the effective rate of acupuncture on both Cuanzhu acupoints of patients with secondary intractable hiccups after traumatic brain injury, was 93.18%, which was significantly better than that (69.05%) in the control group (using chlorpromazine).^[23]^ Although acupressure or acupuncture combined with other techniques has been widely used to treat intractable hiccups, there are no standardized treatments in the clinic. In addition, there is a lack of more objective-controlled studies comparing the various methods, due to which we cannot judge the difference in efficacy between the various methods.

## 2 Objective

The purpose of this systematic review is to conduct a meta-analysis to evaluate the efficacy and safety of acupuncture at acupoints dominated by Cuanzhu (BL2) for controlling intractable hiccups using a meta-analysis of RCTs.

## 3 Methods

### 3.1 Design

A systematic review and meta-analysis will be carried out in this study, which will only include RCTs and will exclude equivalence trials and clinical inferiority trials.

### 3.2 Registration

We have registered the protocol on the International Prospective Register of Systematic Reviews (PROSPERO) (registration number: CRD42020114900). This study will be conducted by strictly following the requirements of the Preferred Reporting Items for Systematic Reviews and Meta-Analyses (PRISMA) guidelines.^[24]^ The summary of this study will follow the PRISMA for Protocol (PRISMA-P) guidelines.^[25]^

### 3.3 Inclusion criteria

This study strictly follows the following criteria for inclusion in the literature.

#### 3.3.1 Types of studies

This study will be evaluated and screened according to the research review guidelines, including participants, interventions, comparisons, and outcomes.

Randomized controlled trials (RCTs) that have used acupuncture as a monotherapy or combined with other treatments to treat intractable hiccups will be included in the study. In case any RCT applied an incorrect random approach, then it will not be included. Furthermore, nonRCTs, animal experiments, human cell or tissue experiments, and repeated publication studies will be excluded.

#### 3.3.2 Types of participants

Adult patients aged >18 years who meet the criteria for persistent and intractable hiccup diagnosis will be included, regardless of gender, nationality, and education level (refer to the Diagnostic Standard for the Clinical Diagnosis and Treatment of Digestive System Diseases^[26]^ for intractable hiccups; the hiccups last for more than 1 month. They could have restarted after 30–60 minutes). Patients who do not meet the diagnostic criteria or have received other interventions will be excluded.

#### 3.3.3 Types of interventions

Experimental group: This group will use acupuncture at acupoints dominated by Cuanzhu (BL2) to treat intractable hiccups based on the control group. The acupuncture method, treatment time, and treatment frequency are not limited.

Control group: This group will include a variety of different conventional treatments, such as no treatment, placebo, sham acupuncture, and drugs.

The routine treatment in the RCTs need not be necessarily similar, but the intervention method must be consistent.

Several comparisons will be analyzed as follows:

1. Acupuncture at acupoints dominated by Cuanzhu (BL2) compared with no treatment
2. Acupuncture at acupoints dominated by Cuanzhu (BL2) compared with placebo
3. Acupuncture at acupoints dominated by Cuanzhu (BL2) compared with sham acupuncture
4. Acupuncture at acupoints dominated by Cuanzhu (BL2) compared with drugs.

#### 3.3.4 Types of outcome measures

##### 3.3.4.1 Primary outcome measures

1. The time when the hiccup symptoms disappear after interventions
2. The frequency or some other change in hiccup symptoms after interventions
3. The adverse events produced by the intervention

##### 3.3.4.2 Secondary outcome measures

1. The number of diverse methods of interventions that are needed to be taken to stop the hiccups
2. Secondary adverse events that do not need withdrawal

### 3.4 Information sources

Eight electronic databases, including 4 Chinese databases (CNKI, SinoMed, Wanfang Database, and Chinese Scientific Journal Database (VIP)), 4 English databases (Web of Science, Medline, Embase, and Cochrane Library) will be searched from their date of establishment to September 2020. In addition, gray literature such as conference papers and bibliographic references will be included. The search was independently completed by 2 researchers in September 2020. Both Chinese and English RCTs will be included.

### 3.5 Search strategies

**Figure 2:**
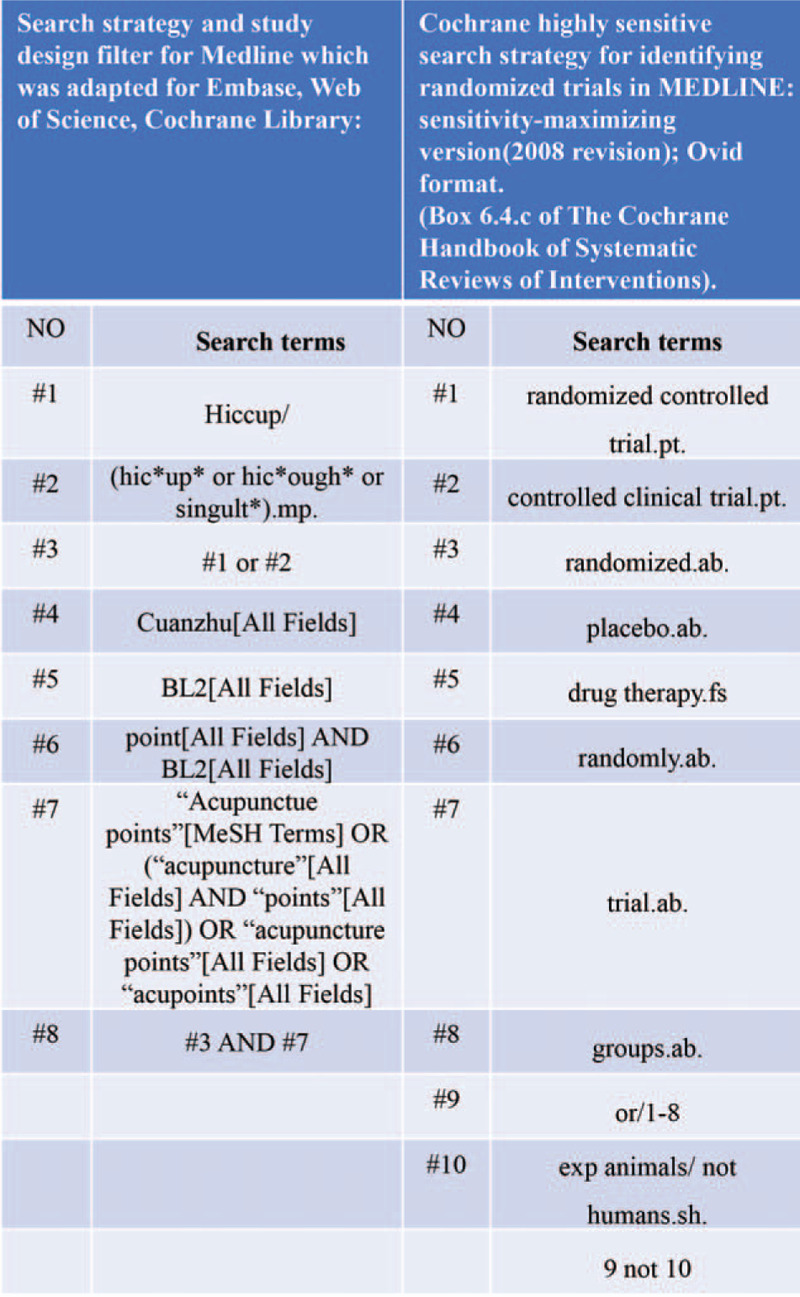
Search strategies.

A comprehensive search strategy will be developed for PubMed or MEDLINE (Fig. 2)

### 3.6 Data collection and management

#### 3.6.1 Selection of studies

**Figure 3:**
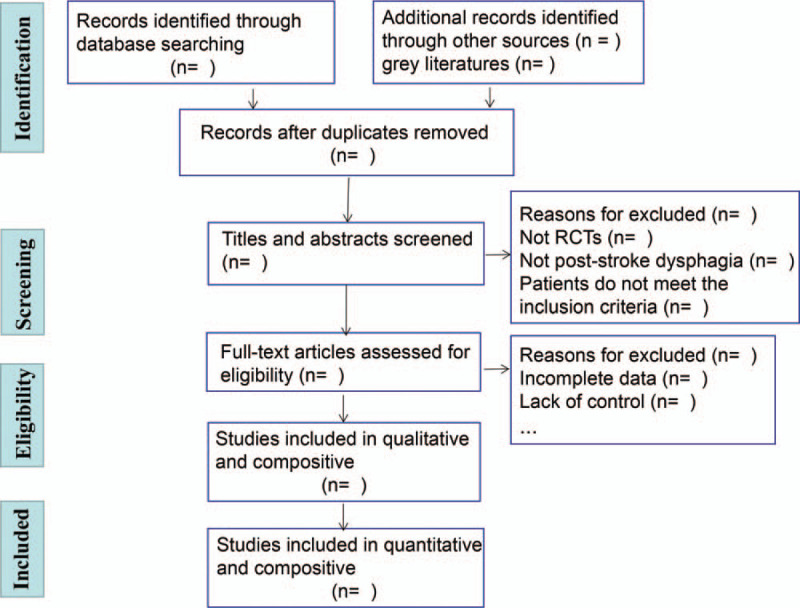
PRISMA flow chart of the study process.

The evaluators are trained and prescreened to ensure the accuracy and standardization of the literature screening process. The literature screening process requires at least 2 reviewers (ZY and WZJ) to conduct independent discussions and discuss the decision with a third commentator (QWX) whenever there is a disagreement. The literature screening process will be reported in detail in the systematic review plan and in the full text. The following steps are included (Fig. 3):

1. Using NoteExpress to classify and organize the initial inspection documents and eliminate duplicate documents
2. Reading the title and abstract of each study and excluding unrelated studies that do not significantly meet the inclusion criteria
3. Analyzing and removing repeated publications
4. Trying to contact the original author to supplement relevant information for studies with incomplete information.

#### 3.6.2 Data extraction

Two reviewers (ZY and WZJ) will use the predesigned standardized information extraction form for extracting research data. The extracted data will include basic information (such as the title, published, journal, publication time, author, ID number, and author contact information,), research methods (such as the inclusion or exclusion criteria, randomization methods, blinding, sample size, intervention group, and control group), intervention cycle, frequency of intervention, participant characteristics (such as age, gender, and grouping,), and other confounding factors (such as the funding source, author key conclusions, author evaluation of confounding factors, and references to other studies, etc.).

### 3.7 Assessment of risk of bias and reporting of study quality

We intend to use the Risk of Bias Assessment tool recommended by the Cochrane Handbook 5.1 to evaluate the methodological quality of the included RCTs.

The following 7 aspects will be included:

1. Random sequence generation (selection bias)
2. Allocation concealment (selection bias)
3. Blinding of participants and personnel (performance bias)
4. Blinding of outcome assessment (detection bias)
5. Incomplete outcome data (attrition bias)
6. Selective reporting (reporting bias)
7. Other bias.

For each of the included studies, an evaluation of “yes” (low bias), “no” (high bias), and “unclear” (indeterminate lack of relevant information or bias) will be done according to the abovementioned 7 terms. The decision will be discussed with the third commentator whenever there is a disagreement. The “Risks of bias graph” and “Risk of bias summary” tools in the RevMan software will be used to represent the percentage of each criterion for all the judged results (“Yes,” “No,” and “Unclear”).

### 3.8 Comprehensive statistical analysis of data

Data analysis and meta-analysis will be performed using the RevMan 5.3 software through the Cochrane collaboration. We will determine the effect size on the basis of different data. The second classification results will be analyzed at a 95% confidence interval (CI) for relative risk (RR) and 95% CI analysis for continuous variables using MD (Standard Deviation).

#### 3.8.1 Handling missing data

If there are incomplete or missing data, we will try to contact the author by e-mail or phone to obtain the most complete data. In case complete data are not available, that particular RCT will be excluded.

#### 3.8.2 Heterogeneity analysis

A Chi-Squared test will be used to estimate the presence of statistical heterogeneity, and the *I*^2^ test will be used to estimate heterogeneity on R-3.3.2 software. If there is no heterogeneity, a fixed effects model (*I*^2^ < 50%, *P* > .05) will be used. In case heterogeneity occurs, a random effects model will be used and a subgroup analysis or sensitivity analysis will be conducted.

#### 3.8.3 Subgroup analysis

If the heterogeneity is obvious, a subgroup analysis will be performed based on different types of acupuncture (such as electroacupuncture, and fire needle,) or other interventions using R-3.3.2 software.

#### 3.8.4 Sensitivity analysis

Sensitivity analysis will be performed to determine data reliability based on missing data, sample size, and heterogeneity using R-3.3.2 software.

#### 3.8.5 Publication bias

A funnel plot will be used to determine if there is a publication bias when there are more than 10 studies included. In case of a publication bias, the funnel plot will appear as an asymmetric distribution.

#### 3.8.6 Grading of evidence quality

The quality evaluation in this study will be completed using the Grading of Recommendations, Assessment, Development, and Evaluation (GRADE) established by the World Health Organization. The quality results will be divided into the following 4 aspects: “high,” “medium,” “low,” and “very low.”

## 4 Discussion

Persistent and intractable hiccups severely reduce the quality of life of patients. In recent years, a large number of studies on pharmacological and nonpharmacological treatments for this disease have been reported. However, there is a lack of a unified diagnostic method and treatment for intractable hiccups in the world. An increasing number of studies have reported that,^[27]^ acupuncture can be used as a type of gastrointestinal response in the treatment of some pathological diseases to improve the quality of life of patients, such as intractable hiccups caused by stroke^[20]^ or postoperative tumors.^[28]^ Acupuncture applied at intractable hiccups points and the methods are diverse, and have obvious advantages of improving the curative effect and shortening the course of the disease.

Cuanzhu (BL2) is an empirical point for acupuncture treatment for intractable hiccups. According to the theory of Traditional Chinese Medicine, Cuanzhu (BL2) belongs to Bladder Meridian of Foot-Taiyang. The bladder meridian passes near the ridge which is connected with the diaphragm, spleen and stomach. Therefore, Cuanzhu (BL2) has the effect of anti-vomiting and stopping hiccups. It was reported that there were facial nerve and pulley nerve branches under Cuanzhu (BL2).^[29]^ After stimulating local nerves, the high-level center of the cerebral cortex could be excited which would inhibit abnormal excitement of the vagus nerve, and alleviate diaphragmatic spasm.^[26,30]^ Cuanzhu (BL2) has been widely used in modern clinical treatment for various types of hiccups caused by encephalopathy, tumors, and other diseases. It is easy to operate and can be activated by pressing, which is suitable for adults and children. However, a systematic review and meta-analysis has not yet been conducted to evaluate the potential benefits and harms of applying acupuncture at acupoints dominated by Cuanzhu in the treatment of intractable hiccups. Therefore, this systematic review is aimed to provide a basis for clinicians to determine the benefits and harms of the treatment of hiccups and evidence for the clinical treatment of hiccups in the field of Chinese medicine, promoting the application of acupuncture at acupoints dominated by Cuanzhu to help more patients.

## 5 Ethics and communication

As this study does not involve raw data collection, no ethical review is required. The results of this study will provide strong evidence for the treatment of intractable hiccups using acupuncture applied at acupoints dominated by Cuanzhu. This will help clinicians to make treatment decisions for intractable hiccups. This systematic review will be published in a peer-reviewed journal and international academic conference.

## 6 Strengths and limitations

This is the first systematic review and meta-analysis to synthesize the utility and safety of acupucturing acupoints dominated by Cuanzhu for improving intractable hiccups. This study can provide a basis for clinicians to determine the benefits and harms of the treatment of hiccups and evidence for the clinical treatment of hiccups in the field of Chinese medicine, improving the quality of life in patients. The methods and quality assessments follow the systematic review guidelines and criterion to minimize the risk of bias. The potential limitation of this study is that only studying the effect of acupoints dominated by Cuanzhu to treat intractable hiccups may cause certain language bias and different types of acupuncture may cause greater heterogeneity.

## Author contributions

**Conceptualization:** Weixin Qin.

**Data curation:** Yu Zhang, Zhijie Wang.

**Investigation:** Yu Zhang, Xudong Jiang, Mingming He.

**Methodology:** Zhijie Wang, Zimeng Lv.

**Project administration:** Xudong Jiang.

**Resources:** Mingming He, Qing Yuan.

**Software:** Yu Zhang, Zimeng Lv, Qing Yuan.

**Writing – review & editing:** Weixin Qin.
